# Influence of 40 Hz and 100 Hz Vibration on SH-SY5Y Cells Growth and Differentiation—A Preliminary Study

**DOI:** 10.3390/molecules27103337

**Published:** 2022-05-23

**Authors:** Patrycja Grosman-Dziewiszek, Benita Wiatrak, Wojciech Dziewiszek, Paulina Jawień, Remigiusz Mydlikowski, Romuald Bolejko, Marta Szandruk-Bender, Ewa Karuga-Kuźniewska, Adam Szeląg

**Affiliations:** 1 Department of Pharmacology, Faculty of Medicine, Wroclaw Medical University, Mikulicza-Radeckiego 2, 50-345 Wrocław, Poland; benita.wiatrak@umw.edu.pl (B.W.); wojciech.dziewiszek@umw.edu.pl (W.D.); paulina.jawien@upwr.edu.pl (P.J.); marta.szandruk-bender@umw.edu.pl (M.S.-B.); adam.szelag@umw.edu.pl (A.S.); 2 Department of Biostructure and Animal Physiology, Wroclaw University of Environmental and Life Sciences, Norwida 25/27, 50-375 Wroclaw, Poland; 3 Faculty of Electronics, Photonics and Microsystems, Wroclaw University of Science and Technology, ul. Janiszewskiego 11/17, 50-372 Wrocław, Poland; remigiusz.mydlikowski@pwr.edu.pl (R.M.); romuald.bolejko@pwr.edu.pl (R.B.); 4 Department of Epizootiology and Clinic of Birds and Exotic Animals, Faculty of Veterinary Medicine, Wroclaw University of Environmental and Life Sciences, 50-366 Wrocław, Poland; ewa.karuga-kuzniewska@upwr.edu.pl

**Keywords:** vibration, low magnitude high-frequency vibration (LMHFV), cell differentiation, cell lines, neurons, SH-SY5Y cells

## Abstract

(1) Background: A novel bioreactor platform of neuronal cell cultures using low-magnitude, low-frequency (LMLF) vibrational stimulation was designed to discover vibration influence and mimic the dynamic environment of the in vivo state. To better understand the impact of 40 Hz and 100 Hz vibration on cell differentiation, we join biotechnology and advanced medical technology to design the nano-vibration system. The influence of vibration on the development of nervous tissue on the selected cell line SH-SY5Y (experimental research model in Alzheimer’s and Parkinson’s) was investigated. (2) Methods: The vibration stimulation of cell differentiation and elongation of their neuritis were monitored. We measured how vibrations affect the morphology and differentiation of nerve cells in vitro. (3) Results: The highest average length of neurites was observed in response to the 40 Hz vibration on the collagen surface in the differentiating medium, but cells response did not increase with vibration frequency. Also, vibrations at a frequency of 40 Hz or 100 Hz did not affect the average density of neurites. 100 Hz vibration increased the neurites density significantly with time for cultures on collagen and non-collagen surfaces. The exposure of neuronal cells to 40 Hz and 100 Hz vibration enhanced cell differentiation. The 40 Hz vibration has the best impact on neuronal-like cell growth and differentiation. (4) Conclusions: The data demonstrated that exposure to neuronal cells to 40 Hz and 100 Hz vibration enhanced cell differentiation and proliferation. This positive impact of vibration can be used in tissue engineering and regenerative medicine. It is planned to optimize the processes and study its molecular mechanisms concerning carrying out the research.

## 1. Introduction

Vibrations play an important role in our lives these days. Their impact can be both positive and harmful. Important parameters are the frequency of vibrations and their proximity depending on the tissue/organ. Despite using vibration in clinical practice, its significance and influence on the human body are still not fully understood. Whole-body or organ vibration is commonly used. Brain stimulation pulses can improve motor skills as well as reduce memory impairment. [1,2]. In addition, the beneficial effect of vibrations supporting the regeneration of muscle and bone tissue is known [3]. The best research was on the effect of vibration on muscle and bone tissue, much less on nervous tissue. The latest works focus on the effects of vibration on the nervous system. These results suggest that vibrations may positively affect mood, the autonomic nervous system, cognitive function, and brain functions [4].

Low-magnitude low-frequency (LMLF) vibration as a non-invasive biophysical intervention includes values of magnitude under 1.0 g, where the acceleration of gravity g = 9.8 m/s^2^ and frequency between 20–100 Hz [1,5]. In vivo and in vitro trials have shown that vibrations like LMLF exert anabolic effects on skeletal metabolism, accelerate fracture and wound healing, and prevent atrophy in musculoskeletal tissues [5,6,7,8,9,10,11]. The combination of thermal images and vibration leads to preulcerative lesions in the diabetic foot [12,13]. Moreover, local vibrations improve microcirculation in diabetic foot ulcers [14]. As an advanced technology, vibration therapy increase wound and fracture healing [15,16]. At present, non-healing wounds and pressure ulcers are serious problems among chronically ill people. Whole-body vibration (WBV) at 45 Hz seems to be promising in this regard. This treatment decreased neutrophil infiltration, and TNF-α levels enhanced collagen deposition in wounds and increased angiogenesis [17,18]. The combination of thermal images and vibration leads to preulcerative lesions in the diabetic foot [12,13].

The low-magnitude, high-frequency mechanical vibration has anabolic potential and stimuli trabecular bone in children. The mechanism of this stimulation is probably the mimic muscular activity in the disabled. Over the course of a longer treatment period, harnessing bone’s sensitivity to these stimuli may provide a non-pharmacological treatment for bone fragility in children [19]. In addition, vibrations may play an important role in neurorehabilitation, enhancing muscle strength and bone mineral content and density [14].

A novel approach for vibrations is still being investigated. Pulses with a frequency lower than 20 Hz (infrasound) can improve the effectiveness of cancer treatment—the mechanism of infrasound on cancer is based on the permeabilization of the cell membrane and sensitization to chemotherapy. Moreover, vibration may demonstrate different therapeutic effects without chemotherapy and depend on the cancer cell type, vibration frequency and time of exposure [20].

The World Association of Vibration Exercise Experts (WAVEX) recommends WBV exercise in patients suffering from a mild COVID-19 infection. This method may reduce fatigue and the risk of dyspnea. WBV exercise also improves the inflammatory and redox status [21]. An example of the clinical use of vibration is respiratory physiotherapy with autonomous vibration belts in critically ill patients with COVID-19 infection with mechanical ventilation in the prone position [22].

We have compiled the vibration research and clinical trials in Table 1. Over a thousand registered clinical trials concern the clinical application of vibration, more than 200 of which have active or recruiting status. The most promising results were obtained using vibrations and low frequencies—40 Hz [2,23,24,25,26,27]. We selected 100 Hz vibrations in the second range, which could also have an adverse effect on the nervous tissue, which is much more sensitive to damage than the bone or muscle tissue and has less regeneration capacity [28,29,30].

On the other hand, the technology improvement makes vibration a universal hazard. Nerves are structures that may be damaged by vibrations, e.g., nerve inflammation in vibration disease [40,41]. Discomfort and even injuries result from vibrations from machines to the human body. Moreover, vibrations may cause damage to the circulatory system, nerves, bones and joints [42]. An example of an effect on nervous tissue might be 250 Hz vibration exposure that may induce oxidative stress in the peripheral nerve and the dorsal root ganglia associated with inflammation [41]. Another example of the negative impact of vibration on the central nervous system could be the pathogenesis of vibration syndrome [40].

With the current technology development, newborns and children may be particularly exposed to the negative effects of vibration because they experience intensive development of the nervous system and the predominance of catabolic processes. Vibration is a physical stressor in neonates during transport and might be associated with increased morbidity, mortality and brain injury [43,44].

Assessment of the impact of vibration on the development and differentiation of nervous tissue is particularly important due to the growth of nervous tissue in children exposed to beat from the first days of life (neonatal transport in trolleys) and in daily contact with vibrations during transport, phone use or in high-tech life.

In this study, we initially investigated the influence of vibration on the development of nervous tissue at the cellular level. Then, a novel nano-vibration system was designed to understand the role of vibrations in neural cell development. Cells, including stem cells of the musculoskeletal tissue, are sensitive to high-frequency, low-intensity mechanical signals. This sensitivity can be utilized for the targeted treatment of tissues and for stem cell expansion, differentiation, and biomaterial interaction in tissue engineering applications [45,46,47].

Vibrations also apply in regenerative medicine in bioreactors that mimic the natural environment for cell culture or stimulate cell differentiation [14,15,48,49]. Previous research findings showed that vibrations could affect stem cell behaviors and lead to the production of mature tissue [6].

SH-SY5Y neuroblastoma cell line has been widely used as an in vitro model for neurotoxicity testing and neurodegenerative disorders treatment model. The SH-SY5Y cells can be differentiated into neurons [50,51]. The selected cell line SH-SY5Y is an experimental research model for Alzheimer’s and Parkinson’s disease [14,15,52,53]. Similar neuronal-like cell line—PC12 cells are involved in a trial for potential biomaterials applications in traumatic brain injury and neural tissue regeneration [54]. Neural-like cell culture might be a previous model for central nervous system activation of neural stem and progenitor cells. These cells have a great regenerative capacity [55]. Human-induced pluripotent stem cells are a great model, but their generating is limited [56]. Our experiment attempts to mimic the neural microenvironment that might support neuronal network creation.

Our work aimed to test the influence of vibration on the nervous tissue and determine the vibration parameters favorable for the growth of nerve cells. We evaluated if low-magnitude low-frequency vibrations (LMLF) 40 Hz and 100 Hz could enhance the neuritis growth and differentiation of the neuroblastoma SH-SY5Y cells. We join biotechnology and advanced medical technology in this research to design the nano-vibration system to produce nanometer-scale vibration on 24-well plates. A scanning laser vibrometer monitored the frequency and amplitude of the nano-vibrations. In addition, the vibration stimulation of SH-SY5Y cell’s growth, differentiation and elongation of their neuritis were observed.

The key question of this paper is if the LMLF vibrations can improve the repair efficiency of nerve cells in tissue engineering. Therefore, optimization of vibration parameters also was the subject of this research. To investigate whether the vibrations can find application in nerve tissue engineering, we measured how vibrations affect nerve cell morphology and differentiation in vitro.

The studies presented in the introduction show a great interest in the clinical application of vibration. However, little is known about LMLF vibration’s influence on neuronal and neuroblastoma cells. Furthermore, the molecular mechanism of the action of vibrations, especially in terms of the influence on the nervous system, is still not understood.

We analyzed the effects of vibration on SH-SY5Y cells in media for differentiation of cells containing retinoic acid and the pulses themselves in a medium containing no retinoic acid. We will assess the vibrations themselves enhance the differentiation of SH-SY5Y cells and improve cells to start forming the morphology of neurons.

## 2. Results

**Figure 1:**
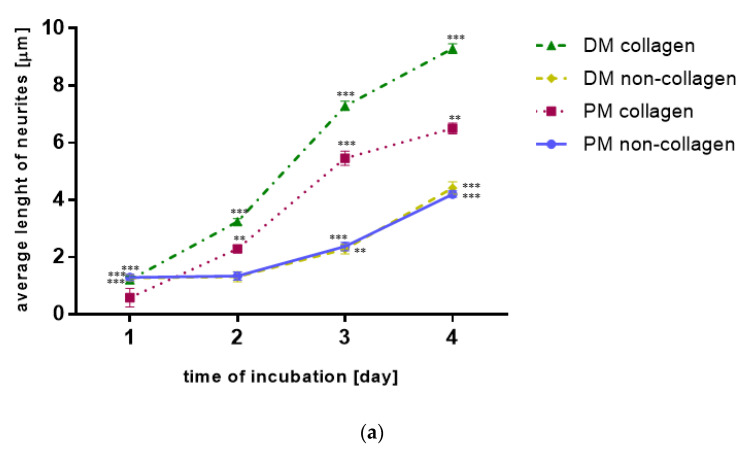

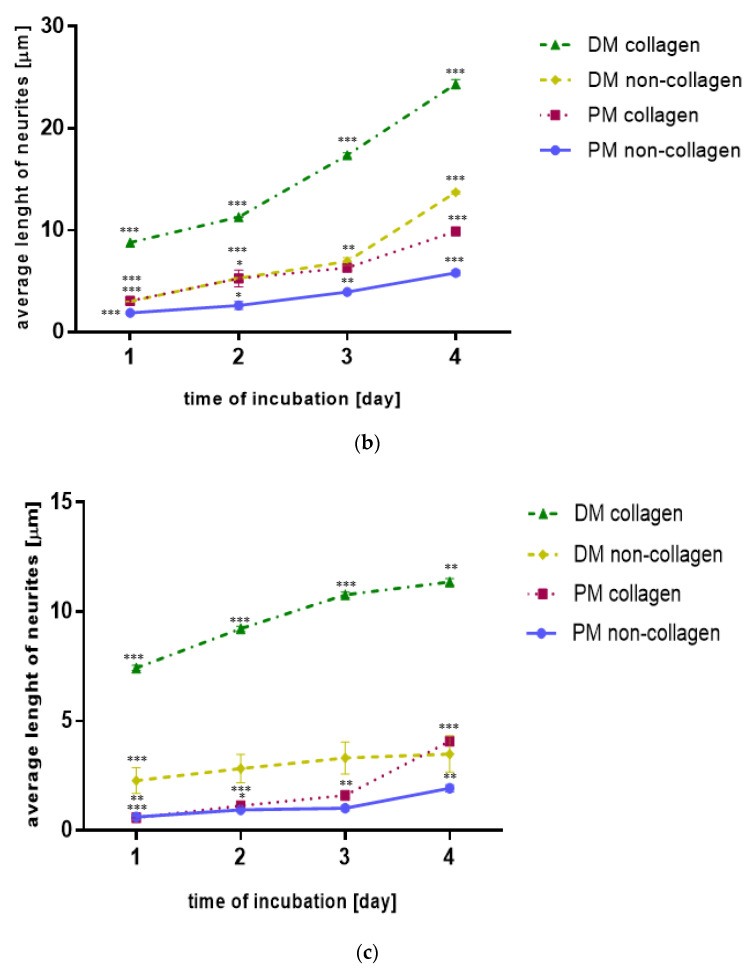
(**a**) The influence of the medium type and the addition of collagen on SH-SY5Y cells neurites length. Results are presented as mean values ± SD. Differences *** *p* < 0.001; ** *p* < 0.01 were deemed statistically significant. (**b**) The influence of vibration with frequency of 40 Hz, the medium type and the addition of collagen on SH-SY5Y cells neurites length. Results are presented as mean values ± SD. Differences *** *p* < 0.001; ** *p* < 0.01, * *p* < 0.05 were deemed statistically significant. (**c**) The influence of vibration with frequency of 100 Hz, the medium type and the addition of collagen on SH-SY5Y cells neurites length. Results are presented as mean values ± SD. Differences *** *p* < 0.001; ** *p* < 0.01, * *p* < 0.05 were deemed statistically significant.

**Figure 2:**
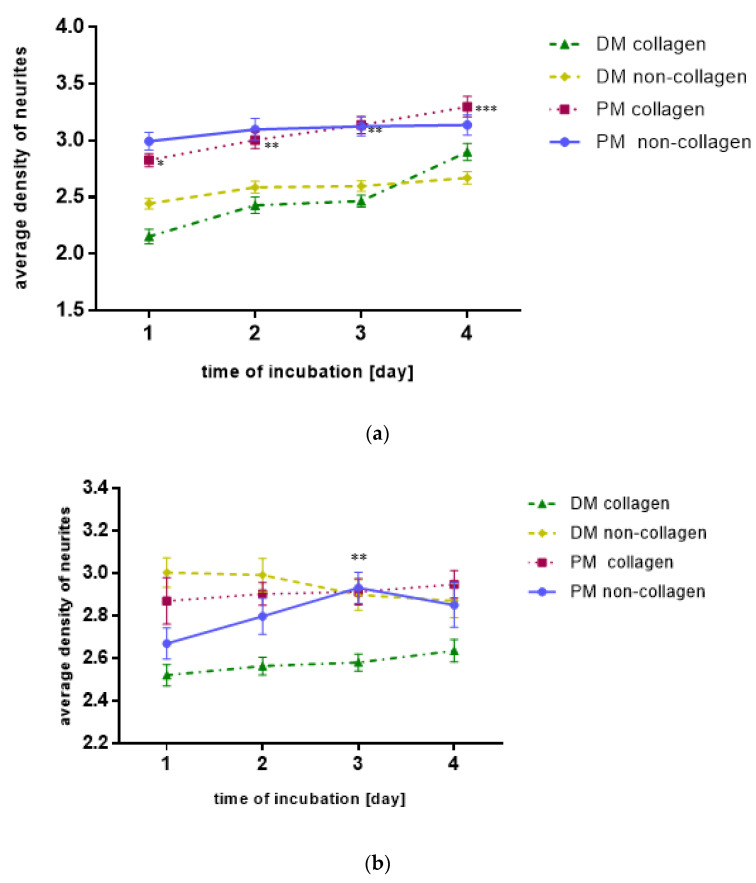

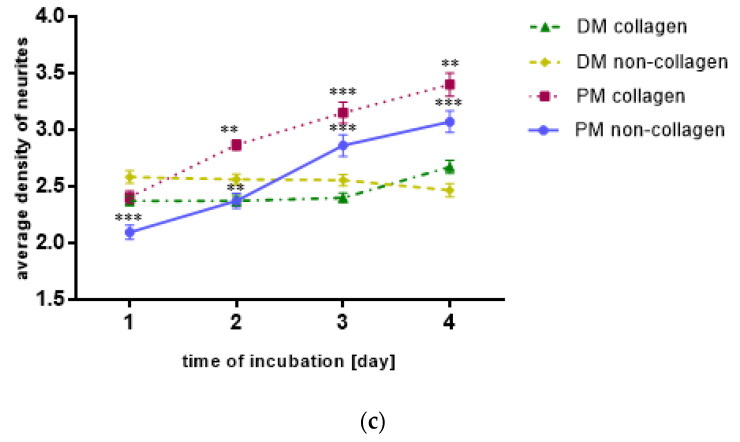
(**a**) The influence of the medium type and the addition of collagen on neurites density of SH-SY5Y cells. Results are presented as mean values ± SD. Differences vs. the previous day *** *p* < 0.001; ** *p* < 0.01; * *p* < 0.1 were deemed statistically significant. (**b**) The influence of vibration with frequency of 40 Hz, the medium type and the addition of collagen on neurites density of SH-SY5Y cells. Results are presented as mean values ± SD. Difference vs. the 1st day ** *p* < 0.01 was deemed statistically significant. (**c**) The influence of vibration with frequency of 100 Hz, the medium type and the addition of collagen on neurites density of SH-SY5Y cells. Results are presented as mean values ± SD. Differences vs. the previous day *** *p* < 0.001; ** *p* < 0.01 were deemed statistically significant.

To assess the impact of vibration with a frequency of 40 Hz or 100 Hz on SH-SY5Y cells morphology and differentiation, the average length of neurites and the average density of neurites (average number of neurites per cell) were measured (Figure 1 and Figure 2).

### 2.1. The Neurites Length

Overall, the neurite length increased significantly when the vibration frequency was 40 Hz than unstimulated cells (*p* < 0.01). Cell’s response did not increase with vibration frequency. The neurites rise after stimulus frequency of 100 Hz was not greater in comparison to unstimulated cells (*p* = NS) and smaller than after stimulus frequency of 40 Hz (*p* < 0.05). The neurite length increased significantly with time for both stimuli frequencies when using the primary or differentiating medium.

Comparing, in detail, the impact of vibration in different groups over time, it was observed that even between days 1 and 2 of the study, there was an increase in neurites length on the non-collagen surface when vibration with a frequency of 40 Hz or 100 Hz was used, while the unstimulated SH-SY5Y cells showed no neurites growth on the non-collagen surface between days 1 and day 2 of the study. The differences were found in neurites growth depending on whether the surface was modified with collagen. The highest average length of neurites was observed in response to the 40 Hz vibration on the collagen surface in the differentiating medium, which was statistically significantly greater than the primary medium with collagen use at the same vibration frequency (*p* < 0.05; Figure 1b). Similarly, the neuron growth was greater on the collagen surface in the differentiating medium than in the primary medium when the vibration frequency was 100 Hz (*p* < 0.001; Figure 1c). There was no difference in neurites length in cultures on the collagen surface between the differentiating medium and primary medium in the unstimulated SH-SY5Y cells.

Moreover, when the vibration frequency was 100 Hz, the neurite length was greater on the collagen surface in the differentiating medium than on the non-collagen surface in this medium (*p* < 0.001; Figure 1c). In the cases of SH-SY5Y cells subjected to stimulation with vibrations at a frequency of 40 Hz and unstimulated SH-SY5Y cells in differentiating medium, modifying the surface with collagen no longer increased the length of neurites (*p* = NS; Figure 1b). In turn, modifying the surface with collagen did not raise the length of neurites in the primary medium, neither for SH-SY5Y cells stimulated by vibrations with the frequency of 40 Hz or 100 Hz, nor for unstimulated SH-SY5Y cells (*p* = NS; Figure 1).

### 2.2. The Neurites Density

In general, vibrations at a frequency of 40 Hz or 100 Hz did not affect the average density of neurites (average number of neurites per cell) compared to unstimulated cells (*p* = NS). However, in contrast to the average length of the neurites, the average number of neurites in response to the 40 Hz vibration on the collagen surface in the primary medium was statistically significantly greater in comparison to the differentiating medium with the use of collagen at the same vibration frequency (*p* < 0.001).

Comparing, in detail, the impact of vibration in different groups over time, it was observed that, on day 3 of the study, there was an increase in the average number of neurites on the non-collagen surface when vibration with a frequency of 40 Hz was used as compared to day 1 of the study (Figure 2b). When vibration frequency was 100 Hz, the neurites density increased significantly with time both for cultures on collagen and non-collagen surface (*p* < 0.001, *p* < 0.001, *p* < 0.01, respectively, in both cases; Figure 2c). In the case of unstimulated SH-SY5Y cells, the neurites density was greater in the primary medium than in the differentiating medium, both for cultures on collagen and non-collagen surface (*p* < 0.05 and *p* < 0.001, respectively). Additionally, in the primary medium, the number of neurites for cultures on the collagen surface increased in the following days of incubation (*p* < 0.001, *p* < 0.01, *p* < 0.01, respectively, Figure 2a).

**Figure 3:**
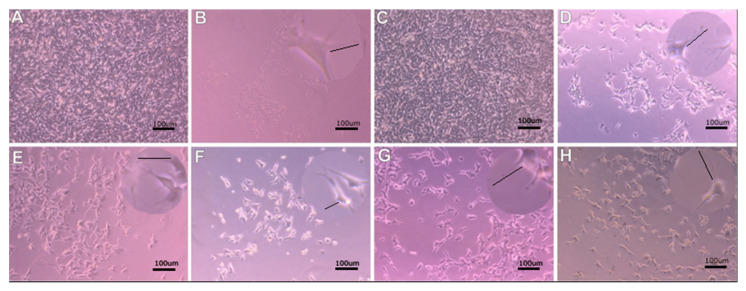
Vibration with a frequency of 40 Hz. (**A**) PM non-collagen—40 Hz, (**B**) PM non-collagen—control, (**C**) PM collagen—40 Hz, (**D**) PM collagen—control, (**E**) DM non-collagen—40 Hz, (**F**) DM non-collagen—control, (**G**) DM collagen—40 Hz, (**H**) DM collagen—control. In the upper right corner, zoom in to show neurites.

**Figure 4:**
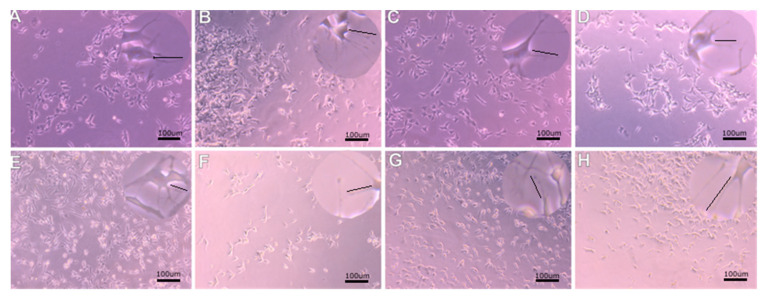
Vibration with a frequency of 100 Hz. (**A**) PM non-collagen—100 Hz, (**B**) PM non-collagen—control, (**C**) PM collagen—100 Hz, (**D**) PM collagen—control, (**E**) DM non-collagen—100 Hz, (**F**) DM non-collagen—control, (**G**) DM collagen—100 Hz, (**H**) DM collagen—control. In the upper right corner, zoom in to show neurites.

Higher SH-SY5Y cell proliferation under 40 Hz vibration was observed (Figure 3). Faster cell growth and greater increase were observed during stimulation with 40 Hz vibrations (Figure 3A,C,E,G) than in the control group, not subjected to vibration (Figure 3B,D,F,H) under the same conditions. Increased neurites length was observed with 40 Hz vibration frequency compared to unstimulated cells (*p* < 0.01) (Figure 3). Neurite length did not increase in 100 Hz vibration frequency (Figure 4).

**Figure 5:**
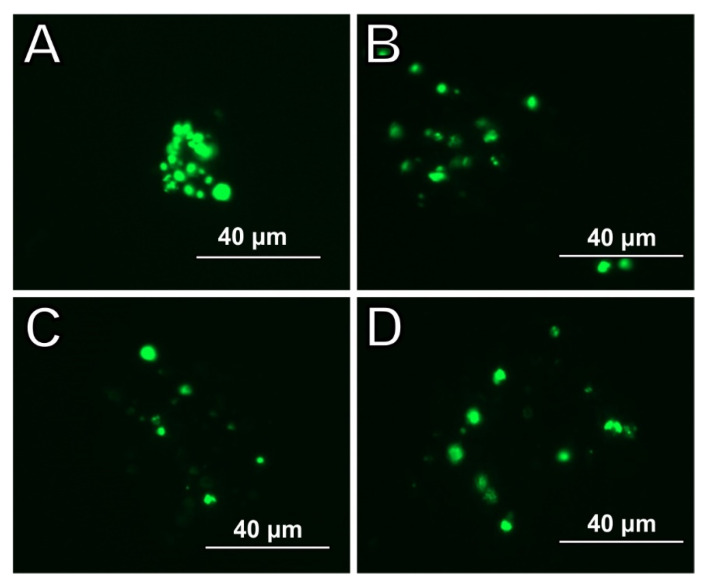
Expression of NeuN after 5 days of treating with frequencies (**A**,**B**) 40 Hz and (**C**,**D**) 100 Hz; (**A**,**C**) non-collagen; (**B**,**D**) collagen.

The expression of NeuN, a marker of differentiated neurons, was assessed after 5 days of vibration therapy. Characteristic expression in cell nuclei. In the cultures incubated in the primary medium, expression was low only at long exposure times (data not shown). On the other hand, in cultures incubated in a differentiation medium, the expression was comparable regardless of the frequency of vibrations used and the modification of the surface of the wells Figure 5.

## 3. Discussion

To our knowledge, this is the first study with acoustic vibration stimulation on the SH-SY5Y cell line. Our research shows that vibration can stimulate the growth and differentiation of SH-SY5Y cells and can find application in tissue engineering. We demonstrate that vibration stimulation with a frequency of 40 Hz of SH-SY5Y human neuroblastoma cells stimulates cell growth and differentiation more than the 100 Hz vibration. The data indicate that the neurite length increased significantly when vibration frequency was 40 Hz than unstimulated cells. Moreover, the nano-vibration system we designed is a reliable experimental model for studying neural-like cell response to vibration.

This paper is one of the first to investigate the effects of 40 Hz and 100 Hz vibration on neural-like cells. Similar studies on SH-SY5Y cells and vibration were performed after electromagnetic stimulation. It was reported that exposure to a 50 Hz electromagnetic field higher than 0.8 mT SH-SY5Y neuroblastoma cells significantly reduced cell viability and mitochondrial transmembrane potential [57,58]. Contrary to our findings, exposure to a 50-Hz magnetic field does not cause proliferation change and apoptosis activation in both SH-SY5Y cultures [59], but 50 Hz sinusoidal magnetic fields caused an intensity-dependent reduction in nerve growth factor stimulation neurite outgrowth in PC-12 cells [60]. No effects were found in nerve cells, but the conductance of gap junction channels was decreased under 60 Hz electromagnetic field exposure. Moreover, the intracellular Ca^2+^ at current densities was significantly increased [61]. However, due to the different types of vibrations used in the presented experiments, these results cannot be directly compared with each other.

Analyzing presented results with published ones, some indirect comparison can be made by considering published reports related to the vibration-induced SH-SY5Ycell differentiation. There are many trials concerning both the in vivo and in vitro use of vibration, mostly in field of bone and muscle cells. However, only a few experiments describe the influence of vibration on neuronal-like cell lines. For example, 10 Hz vibration promoted PC12 cells exposed to nerve growth factor to differentiation and elongation of their neurites [62]. Furthermore, it was reported that 10,20,30 and 40 Hz vibration 5 days of stimulation of umlimbial cord stem cells (hUC-MSCs) shows the neurogenic effect and increased expression of MAP2, NF-L and Neuro D1 [63,64,65].

Our study measured the impact of vibration with a frequency of 40 Hz or 100 Hz on SH-SY5Y cells morphology and differentiation of the average length and density of neurites. We found that our results with the frequency of 40 Hz agree with results that show neurite elongation in 50 Hz stimulation neuronal-like PC-12 cells given in [62]. An interesting discovery was the increased SH-SY5Y cell proliferation in the differentiation medium (Figure 5c). The highest average length of neurites responded to the 40 Hz vibration on the collagen surface in the differentiating medium (Figure 5a). However, these results were not reflected in neurites density, which requires further research to determine the molecular mechanisms of vibrations influence (Figure 5a,b). The addition of retinoic acid to the cell culture medium inhibits growth cells and cellular differentiation-promoting properties. SH-SY5Y neuroblastoma cells differentiate into more mature, neuron-like phenotype cells with increased electrical excitability of the plasma membrane and synaptophysin-positive functional synapses. Differentiated SH-SY5Y cells do not cluster and have a more pyramidal-shaped cell body (Figure 3 and Figure 4). The undifferentiated SH-SY5Y cells characterized neuroblast-like, non-polarized cell morphology and continuously increased [61]. Differentiated SH-SY5Y cells are withdrawn from the cell cycle and decreased proliferation [66,67]. Moreover, the differentiation medium activates the phosphatidylinositol 3-kinase/Akt signaling pathway. It upregulates the antiapoptotic Bcl-2 protein, expressing many mature neuronal markers, including βIII-tubulin, microtubule-associated protein-2 (MAP2), synaptophysin, and neuronal nuclei (NeuN), synaptic associated protein-97 (SAP-97), and differentiation-promoting genes NEUROD6 and NEUROD1 [68,69]. Our data shown characteristic expression of NeuN marker of differentiated neurons in cell nuclei in the cultures incubated in a differentiation medium. This finding confirm that the expression was comparable regardless of the frequency of vibrations but requires further study.

In vivo and in vitro trials have shown that vibrations exert anabolic effects on skeletal metabolism by promoting osteogenic activity, accelerating fracture and wound healing, preventing disuse atrophy in musculoskeletal tissues, improving the bone structure and muscle performance in a variety of different patient groups and could enhance chondrogenic differentiation of human adipose-derived mesenchymal stem cells [14,15,16,17,70]. Vibration has promising applications in many various diseases. However, the mechanism of action is still poorly understood.

To study cell behavior under in vitro conditions, it is important to mimic the dynamic environment of the in vivo state. Robertson et al. described a novel bioreactor platform of cell cultures using nanovibrational stimulation to discover the field of gravitational wave astronomy [48]. A similar experiment but with cardiac muscle cells was performed. To measure cardiac muscle HL1 cells, cytoskeletal changes in response to different sounds cells were exposed to 20-min sound sequences in a specially designed environment. This trial concluded that acoustic waves affect cytoskeletal molecules and change mechano-transduction signaling [71]. A 2-way connected culture of human neuroblastoma cells SH-SY5Y and human coronary artery smooth muscle cells HCASMC—LiveBox2 bioreactor was developed [72]. Phonomimetic bioreactor was invented to examine the human vocal folds fibroblasts under inflammatory conditions and vocal fold diseases treatment with regenerative medicine and tissue engineering application [9,10]. A nanovibrational bioreactor for controlled osteogenesis of MSCs without chemical induction was also designed [15,69,73]. This bioreactor system [30] requires mechanical stimulation with a frequency of 1 kHz for osteogenesis. In our experiment, neuronal-like cells need 40 Hz vibration for growth and differentiation stimulation. Bioreactor with neural-like PC12 cells demonstrates the beneficial effects of combining physical stimuli in cell culture systems to promote neurogenesis [74].

The molecular mechanism of vibration is still unclear and based on the influence on gene and protein expression. However, the impact of LMHFV improved secretion of Bone Morphogenetic Protein 2 (BMP-2), Collagen type II, with a low concentration of Collagen type I and upregulated Sox-9, α3, α4, β1 and β3 integrins gene expression in chondroblast progenitor cells [10]. In addition, LMHFV induced bone marrow stem cell osteogenic differentiation and promoted bone formation via the Wnt signaling pathway upregulated miR-335-5p, which suppresses the expression of the Wnt signaling inhibitor Dickkopf-related protein 1 [48].

Recent studies show that oxygen shortage and hypoxia shifts neuroblastoma cells into stem cell-like phenotypes [69]. Perhaps the cells exposed to the vibrations became neoplastic. Faster cell growth was observed in the group exposed to 40 Hz vibration. Research should be carried out on primary lines, which are the best marker of the nervous system.

Preclinical and clinical studies report the impact of vibration in Alzheimer’s disease pathology on human and animal models. Vibration therapy improves physical and cognitive deficits [75]. The mechanism of this impact is the reduction of amyloid-β in plaques in the hippocampus and entorhinal cortex [76,77] and the decrease of *p*-Tau levels in the hippocampus [78,79]. The other mechanism of vibrations is the modulation of brain network activity [26,80,81,82] and neurogenesis and dendritic growth in the hippocampus [67,69]. The clinical pilot study confirmed that stimulation at 40 Hz with sound stimuli improved patients’ cognition with Alzheimer’s disease [25,26,83].

Concerning clinical and preclinical studies, we can confirm the use of vibration in the stimulation and regeneration of the nervous system. The obtained results show the beneficial effects of vibration at the cellular level. We assessed that 40 Hz and 100 Hz vibrations are safe for neuronal-like SH-SY5Y cells in 5 day/8 h cycle. That may suggest that vibrations in this range will not negatively affect the rehabilitation. A specially designed nano-vibration system will also allow learning about the effects of pulses at the molecular level.

## 4. Materials and Methods

### 4.1. Cell Line and Conditions

The SH-SY5Y cells were used in this study at 9–14 passages. These cells were purchased from ATCC and grown at 37 °C, 5% CO_2_, and 95% humidity with recommended medium by ATCC. (1:1 mixture of Eagle’s Minimum Essential Medium from Biological Industries, Kibbutz Beit Haemek, Israel and F12 Medium from Biological Industries Kibbutz Beit Haemek, Galilee, Israel). This medium was supplemented with 10% Fetal Bovine Serum (FBS—from Biological Industries, Kibbutz Beit Haemek, Israel) and 2 mM L-glutamine (Lonza company, Basel, Switzerland), 1.25 µg/mL amphotericin B (Gibco company, Grand Island, New York, NY, USA), and 100 µg/mL gentamicin (Gibco company, Grand Island, New York, USA)—primary medium. The second medium for differentiation of cells was the same mixture, but the FBS was reduced to 2.5%, and 10 µM retinoic acid (Sigma-Aldrich R2625, Saint Louis, MO, USA) was added [64].

### 4.2. Experimental Design

**Figure 6:**
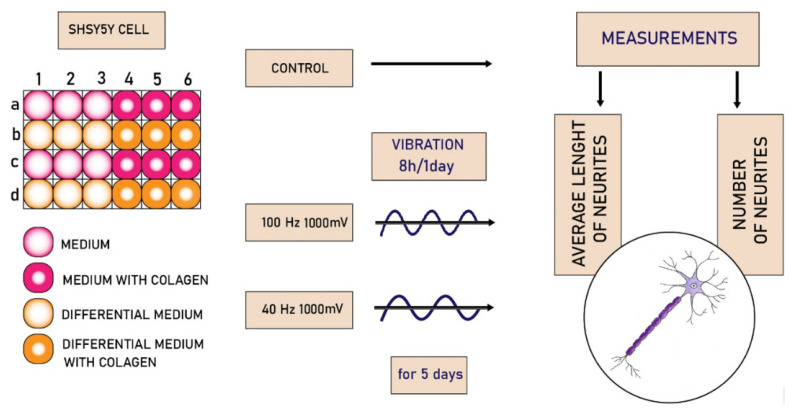
Block diagram of the medical experiment carried out.

The biological experiment was performed on 24-well plates. A half of all wells were modified using collagen type I (Sigma-Aldrich, Saint Louis, USA). The modification was performed as previously described [43]. The cells were seeded on a whole plate and incubated overnight to allow the cells to adhere to the surface of the wells. The next day, the medium was removed, the cell differentiation medium was added to half of the wells, and the remaining wells were supplemented with the primary medium (Figure 6).

**Figure 7:**
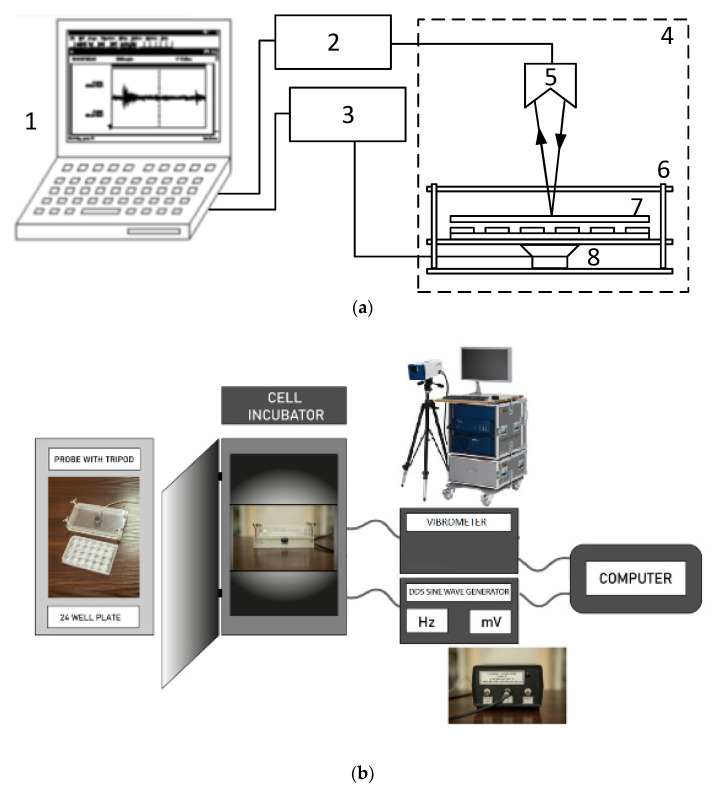
(**a**) Laboratory stands for the generation and measurement of nanoscale vibrations. 1—computer, 2—vibrometer, 3—DDS sine wave generator, 4—incubator, 5—laser vibration detector, 6—plexiglass enclosure, 7—24 cell-culturing plate, 8—low-frequency loudspeaker. (**b**) Cells were placed on a probe with a tripod in the CO_2_ incubator.

The experiment was conducted on a test stand built for this research (Figure 7a). During the investigation, LMLF vibrations with frequencies of 40 and 100 Hz and amplitude of 1 VRMS were induced. Such a selected amplitude of the generator of sinusoidal signals was chosen to obtain a sufficiently large distance of the measured values of vibrations of the tested plate from the so-called background. The background is understood to be the naturally occurring vibrations in the laboratory room. The signal set in computer 1 was supplied by the sine wave generator DDS 3 to power the low-frequency speaker 8. The DDS generator used during the measurements is characterized by a constant frequency value in time, which was important for the long time of the measures (8 h/day). The low-frequency loudspeaker 8, working in the frequency range of (10–300) Hz, was permanently connected to the Plexiglass enclosure 6. A measuring plate 7 with 24 cells was placed in the enclosure. The plate was subjected to mechanical vibrations generated by the loudspeaker at a defined time during the day (8 h/day). The vibration level of each plating cell was measured in the direction perpendicular to the plate surface using a scanning laser vibrometer 5 type Polytec PSV400M2.

**Figure 8:**
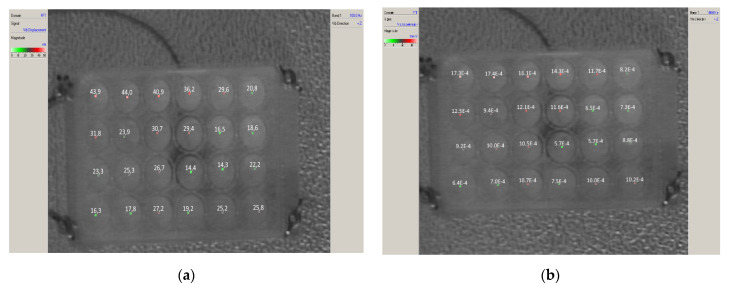
Distribution of vibrations of individual cells of the plate for f = 100 Hz; (**a**)—vibration amplitude of plate cells [nm], (**b**)—acceleration of plate cells [mm/s^2^].

Figure 8 showed the distribution of vibrations of particular cells of the measuring plate when the sounder was excited by the signal of frequency 100 Hz. Figure 8a presents the obtained displacements in the y-axis (vibration amplitudes) in nm, and Figure 8b shows the obtained accelerations $\vec{y}$ in mm/s^2^. The recorded motion of a culture plate can be defined as a course of sinusoidal function with a given frequency and amplitude of acceleration.

(1) y=Asin(ωt) →y→=−ω^2sinα(ωt)

where: y—displacement, $\vec{y}$—acceleration, A—vibration amplitude, ω—vibration pulsation (2πf), f—frequency, t—time, Aω^2—acceleration amplitude.

Vibrations with the highest amplitude of 44 nm were observed in the upper left corner, with the lowest pulses in the opposite corner at 14 nm. They correspond to the obtained accelerations at the level of (1.7–0.6) mm/s^2^. Due to the construction of the test stand, the method of fixing the plate and loudspeaker, and the properties of the material from which the enclosure is made, the “swaying” of the test stand concerning the diagonal: bottom left corner-top right corner occurs. However, small differences in vibration amplitudes (ca. 20–30 nm) and accelerations (ca. 1 mm/s^2^) had no significant effect on the differentiation of the growth of the studied cells over a long time interval. Therefore, it can be concluded that the plate under test at low frequencies (40, 100) Hz vibrates as a hard element with practically no division into smaller fragments. This has to do with the longer length of the resulting mechanical waves at low signal frequencies.

The cells were placed on a probe with a tripod in the CO_2_ incubator (Figure 2b). Every day was set vibration (100 Hz, 1 V or 40 Hz, 1 V) for 8 h. The cells were evaluated under microscopy the next day, and 5 photos were taken from every well. After taking pictures, the DDS sine wave generator was set for the next 8 h.

### 4.3. The Neurites Length and Density Measurement

**Figure 9:**
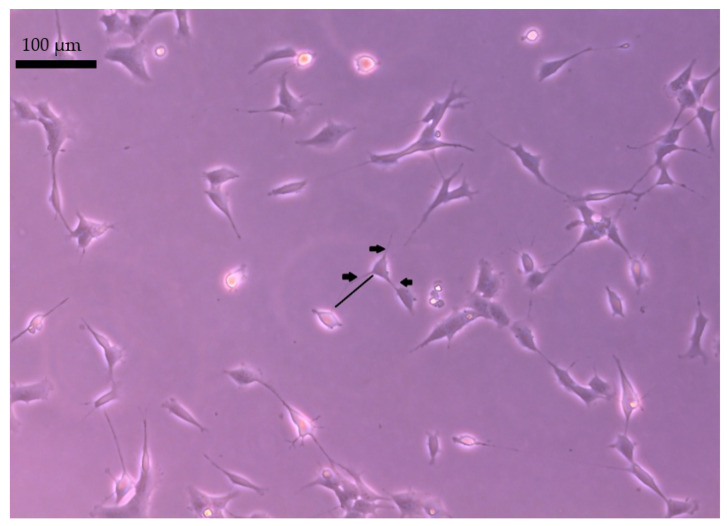
Density and lengths of neurites measurement. The length of neurites is marked with a black line, while the number is marked with arrows.

Two independent observers performed the same procedure of neurites length and density measurement. The average length and density of neurites were evaluated based on microscopic images (Figure 9). Using the ImageJ software, the ImageJ software determined the lengths of 50 cells in 3 different wells in 5 independent replicates. Neurite density is understood as the average number of neurites per cell.

### 4.4. Immunofluorescence Procedure

Cells were fixed with cold methanol for 5 min at room temperature and discarded. Subsequently, cell membrane permeabilization was performed by washing 3 times for 5 min with PBST (0.1% Tween 20 in PBS) and 10 min incubation with 0.1% Triton X-100 solution in PBST for 10 min at room temperature. After washing three more times with PBS, the blocking of nonspecific binding of antibodies was performed in a solution containing 1% bovine serum albumin (BSA) in PBST for 30 min. Cell cultures were then incubated with anti-NeuN antibodies (1: 200; Abcam, Cambridge, UK; Cat # ab6328) and anti-doublecortin (DCX) (1: 100; Sigma-Aldrich; Cat # ab52987) in 1% BSA in PBST at 4 °C overnight After washing thoroughly in PBS, cultures were embedded into mounting medium (3% glycerin in PBS) and examined under EVOS FL fluorescence microscopy.

### 4.5. Statistical Analysis

Two independent researchers measured neurite lengths using the free open space ImageJ and the number of neurites per cell. In addition, 50 neurite lengths were calculated for each well on each day. The procedure of histomorphological evaluation was performed according to the method described by Wiatrak et al. [57]. The sample size includes 6 repetitions for 1 combination (medium, differential medium, with collagen, without collagen) made in 3 repetitions at different times. The collected results were statistically analyzed in Statistica v. 15. Due to a large number of measurements performed, it was not necessary to maintain the normal distribution of the results to be able to calculate statistical significance using parametric tests. Therefore, ANOVA and post-hoc Scheffe analyses were used.

## 5. Conclusions

To summarize, the exposure of neuronal cells to 40 Hz and 100 Hz vibration enhanced cell differentiation and proliferation. The positive impact of vibration can be used in tissue engineering and regenerative medicine. A novel bioreactor platform of neuronal cell cultures using LMLF vibrational stimulation was designed to discover vibration influence and mimic the dynamic environment of the in vivo state.

The most promising finding is that 40 Hz vibration best impacts neuronal-like cell growth and differentiation. Even though in the ranges of higher vibrations (100 Hz), we could expect damage or inhibition of the development of nerve cells, the results obtained by us indicate stimulation of the growth of nerve cells by the applied vibrations. This may mean that vibrations in this range will not negatively affect muscle or bone tissue rehabilitation but may also support the treatment of nervous tissue in various diseases.

The mechanism of this observation needs further molecular investigation to be clearly understood. Nevertheless, the results indicate a greater potential for low-frequency vibrations in tissue engineering for nerve cells.

Further research is planned to optimize the processes in the bioreactor and investigate the molecular mechanisms that occur in nerve cells under the influence of vibrations.

## Figures and Tables

**Table 1:** The vibration research and clinical trials.

Vibrations	Clinical Implication	Participants
100–400 Hz underwater vibrations	Vibrotimulation—produced hearing-like sensations in the ear [28]	adults and children with congenital hearing loss
100 Hz localized vibration	motor recovery in neurorehabilitation [29]	healthy men
100 Hz vibration	flexors spasticity reduction [30]	hemiplegic patients affected by upper limb spasticity
40 Hz 12 weeks of vibration therapy	Parkinson’s disease [2]	Adults with Parkinson’s disease
40 Hz auditory stimulation	Alzheimer’s disease [23]	Alzheimer’s disease mouse models
20-Hz to 40-Hz vertical vibration	increased brain network activity [24]	women with senile dementia
40 Hz acoustic stimulation	Alzheimer’s disease [25]	Alzheimer’s disease mouse models
40 Hz transcranial ultrasound	Alzheimer’s disease [26]	Alzheimer’s disease mouse models
40 Hz sound stimulation	Alzheimer’s disease [27]	persons with mild and moderate Alzheimer’s disease
35 Hz LMHFV vibration therapy	hip fracture healing [31]	Elderly males or females aged 65 years or older with unilateral trochanteric hip fractures
33 Hz vibration platform therapy	spastic cerebral palsy [32]	Children with physical disabilities
90 Hz low magnitude vibrations	treatment for bone fragility in children [22]	children with disabling conditions
12–30 Hz	optimal functional conditions, which may allow the children to be physically active [33]	pediatric cancer patients and survivors
27–32 Hz vibration platform therapy	After vibration training, there was no significant difference between groups for bone resorption [34]	women with breast cancer
35 Hz vibration platform therapy	Enhancing bone and muscle quality [35]	disabled older wheelchair users
12,5 Hz whole-body vibration therapy	improves static balance in patients with fibromyalgia [36]	women with fibromyalgia
30 Hz vertical vibrations	prevention of postmenopausal bone loss [37]	women, 3–8 years past the menopause
whole-body vibration (WBV)	Improved blood pressure in the hypertensive population [38]	hypertensive males and females
Vibration frequencies of 1–8 Hz	Increase LF/HF (the relationship between sympathetic and parasympathetic nerve activities), affected sympathetic activity [39]	healthy young men
8 Hz vibration belt	respiratory physiotherapy with vibration belts [21]	critically ill patients with COVID-19 infection with mechanical ventilation
15 Hz local vibration	plantar blood flow improvement in diabetic foot ulcers [22]	diabetic and healthy adults

## Data Availability

The data is available from the corresponding author.
